# Supplementary material for: Development of a computer-aided design software for the quantitative evaluation of aesthetic damage
Source: PLoS One. 2019 Dec 18;14(12):e0226322. doi: 10.1371/journal.pone.0226322 (PMC6919621; doi:10.1371/journal.pone.0226322)
Supplement: S1 Code site — 1. (ZIP) [file pone.0226322.s001.zip › [EESC_jr][Projeto]Relatorio_07_21/reports/templates_zip/gentelella-master/vendors/morris.js/examples/no-grid.html]

# Formatting Dates YYYY-MM-DD

```
/* data stolen from http://howmanyleft.co.uk/vehicle/jaguar_'e'_type */
var day_data = [
  {"period": "2012-10-01", "licensed": 3407, "sorned": 660},
  {"period": "2012-09-30", "licensed": 3351, "sorned": 629},
  {"period": "2012-09-29", "licensed": 3269, "sorned": 618},
  {"period": "2012-09-20", "licensed": 3246, "sorned": 661},
  {"period": "2012-09-19", "licensed": 3257, "sorned": 667},
  {"period": "2012-09-18", "licensed": 3248, "sorned": 627},
  {"period": "2012-09-17", "licensed": 3171, "sorned": 660},
  {"period": "2012-09-16", "licensed": 3171, "sorned": 676},
  {"period": "2012-09-15", "licensed": 3201, "sorned": 656},
  {"period": "2012-09-10", "licensed": 3215, "sorned": 622}
];
Morris.Line({
  element: 'graph',
  grid: false,
  data: day_data,
  xkey: 'period',
  ykeys: ['licensed', 'sorned'],
  labels: ['Licensed', 'SORN']
});
```
